# Recent advances in central congenital hypothyroidism

**DOI:** 10.1530/JOE-15-0341

**Published:** 2015-12

**Authors:** Nadia Schoenmakers, Kyriaki S Alatzoglou, V Krishna Chatterjee, Mehul T Dattani

**Affiliations:** 1 University of Cambridge Metabolic Research Laboratories, Wellcome Trust-Medical Research Council Institute of Metabolic Science, Addenbrooke's Hospital, Level 4, PO Box 289, Hills Road, Cambridge, CB2 0QQ, UK; 1 Developmental Endocrinology Research Group, Section of Genetics and Epigenetics in Health and Disease, Genetics and Genomic Medicine Programme, UCL Institute of Child Health, London, UK

**Keywords:** congenital hypothyroidism, central hypothyroidism, hypopituitarism, thyrotropin releasing hormone

## Abstract

Central congenital hypothyroidism (CCH) may occur in isolation, or more frequently in combination with additional pituitary hormone deficits with or without associated extrapituitary abnormalities. Although uncommon, it may be more prevalent than previously thought, affecting up to 1:16 000 neonates in the Netherlands. Since TSH is not elevated, CCH will evade diagnosis in primary, TSH-based, CH screening programs and delayed detection may result in neurodevelopmental delay due to untreated neonatal hypothyroidism. Alternatively, coexisting growth hormones or ACTH deficiency may pose additional risks, such as life threatening hypoglycaemia. Genetic ascertainment is possible in a minority of cases and reveals mutations in genes controlling the TSH biosynthetic pathway (*TSHB, TRHR*, *IGSF1*) in isolated TSH deficiency, or early (*HESX1, LHX3, LHX4, SOX3, OTX2*) or late (*PROP1, POU1F1*) pituitary transcription factors in combined hormone deficits. Since TSH cannot be used as an indicator of euthyroidism, adequacy of treatment can be difficult to monitor due to a paucity of alternative biomarkers. This review will summarize the normal physiology of pituitary development and the hypothalamic–pituitary–thyroid axis, then describe known genetic causes of isolated central hypothyroidism and combined pituitary hormone deficits associated with TSH deficiency. Difficulties in diagnosis and management of these conditions will then be discussed.

## Introduction

Central congenital hypothyroidism (CCH) is a rare disorder in which inadequate thyroid hormone biosynthesis occurs due to defective stimulation of a normal thyroid gland by thyroid stimulating hormone (TSH). The underlying molecular basis is often undefined, but hypothalamic or pituitary pathology contributes to a qualitative or quantitative deficit in TSH synthesis or secretion (Persani 2012). In a minority of cases, TSH deficiency is isolated and may occur as a result of defects in genes controlling the TSH biosynthetic pathway, eg mutations in the thyrotropin-releasing hormone receptor (*TRHR*), thyroid stimulating hormone β subunit (*TSHB*) and the more recently-described immunoglobulin superfamily member 1 gene (*IGSF1*) (Garcia *et al*. 2014). Alternatively, as normal pituitary development depends on the sequential temporal and spatial expression of a cascade of signaling molecules and transcription factors, mutations in early (*HESX1, LHX3, LHX4, SOX3, OTX2*) or late (*PROP1, POU1F1*) transcription factors may cause central hypothyroidism with or without associated extrapituitary abnormalities. However, in these cases, central hypothyroidism does not occur in isolation, but is one of the evolving pituitary hormone deficiencies (Alatzoglou & Dattani 2009, Kelberman *et al*. 2009).

Since TSH is not elevated in CCH, this entity will evade diagnosis in TSH-based, primary CH screening programs, and patients are at risk of neurodevelopmental delay if severe CCH remains untreated postnatally. Additionally, the delayed diagnosis of other pituitary hormone deficiencies (Adrenocorticotrophic hormone; ACTH, growth hormone; GH) may pose significant risks, such as life threatening hypoglycaemia.

In spite of current knowledge regarding genetic causes of CCH, the majority of cases do not have an identifiable molecular defect in known causative genes. This review will summarize our current understanding of the molecular genetics underlying CCH as well as highlighting controversies in treatment and diagnosis.

## Physiology/embryology

### Morphogenesis and cellular differentiation in pituitary development

**Figure 1:**
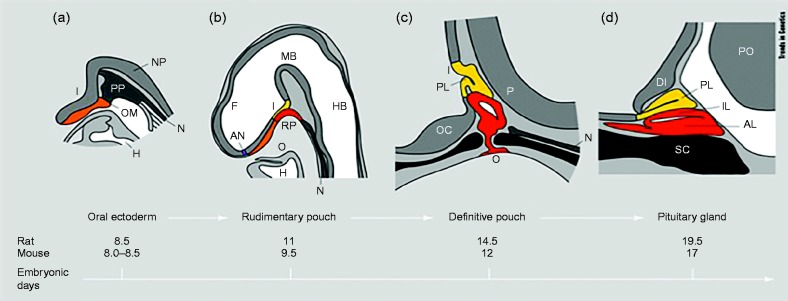
Schematic representation of the stages of pituitary development in rodents: (a) Oral ectoderm (b) Rudimentary pouch (c) Definitive pouch (d) Adult pituitary gland. The close contact between the developing Rathke's pouch (red) and the infundibulum (yellow) is maintained throughout and is important for the normal morphogenesis of the gland. I, infundibulum; NP, neural plate; N, notochord; PP, pituitary placode; OM, oral membrane; H, heart; F, forebrain; MB, midbrain; HB, hindbrain; RP, Rathke's pouch; AN, anterior neural pore; O, oral cavity; PL, posterior lobe; OC, optic chiasm; P, pontine flexure; PO, pons; IL, intermediate lobe; AL, anterior lobe; DI, diencephalon; SC, sphenoid cartilage. Reprinted from *Trends in Genetics*, volume 15, Sheng HZ, Westphal H, Early steps in pituitary organogenesis, pages 236–240, Copyright (1999), with permission from Elsevier.

The development of the anterior pituitary requires the sequential temporal and spatial expression of a cascade of signaling molecules and transcription factors that are important for organ commitment, cell proliferation, patterning and terminal differentiation. Human pituitary gland development in humans largely mirrors that in rodents (Sheng & Westphal 1999) (Fig. 1). The anterior pituitary develops from the hypophyseal placode that appears ventrally in the midline of the anterior neural ridge at embryonic day (E) 7.5, and is in continuity with the future hypothalamo-infundibular region, which is located in the rostral part of the neural plate (Rizzoti & Lovell-Badge 2005). By E8.5 the placode appears as a thickening of the roof of the primitive oral cavity and at E9.0, it invaginates to form the rudimentary Rathke's pouch, from which the anterior and intermediate lobes of the anterior pituitary are derived (Takuma *et al*. 1998, Rizzoti & Lovell-Badge 2005). The definitive pouch is formed by E10.5, whilst the neural ectoderm at the base of the developing diencephalon evaginates to give rise to the posterior pituitary. The pouch epithelium continues to proliferate between E10.5 and E12 and separates from the underlying oral ectoderm at E12.5. The progenitors of the hormone-secreting cell types proliferate ventrally from the pouch between E12.5 and 17.5 to populate the future anterior lobe (Ward *et al*. 2005). Progenitor cells divide around the lumen of Rathke's pouch and relocate ventrally as they differentiate. This ventral relocalisation is associated with exit from the cell cycle (Drouin *et al*. 2010) and expression of cyclin-dependent kinase inhibitor 1C (p57Kip2) and cyclin E, at the boundary between the lumen and the forming anterior lobe (Bilodeau *et al*. 2009).

In the developing pituitary, there are two populations of thyrotrope cells: a transient population of rostral tip thyrotropes and the ‘definitive’ thyrotropes that will populate the anterior pituitary. The earliest marker of differentiation in the anterior pituitary is the expression by E11.5 of αGSU (alpha-glycoprotein subunit; *Cga*) in a restricted patch of cells in the ventral region of Rathke's pouch. These αGSU positive cells will express the transcription factor Islet-1 (*Isl1*) and will differentiate at E12.5 by initiating the expression of thyroid stimulating hormone subunit-β (*Tshb*) (Ericson *et al*. 1998, Kelberman *et al*. 2009). This cell population, referred to as rostral tip thyrotropes, is POU1F1-independent and will disappear at birth (Himes & Raetzman 2009). The definitive thyrotropes are detected later, at E14.5, after the appearance of corticotropes (that start to differentiate at E12.5), as defined by the expression of *Pomc* (Zhu *et al*. 2007, Kelberman *et al*. 2009). This cell population, referred to as ‘thyrotropes’, is POU1F1-dependent and will secrete functional TSH. In the adult pituitary gland, there are no rostral tip thyrotropes and the expression of αGSU will only be detected in thyrotropes and gonadotropes (Ericson *et al*. 1998, Himes & Raetzman 2009, Kelberman *et al*. 2009). The expression of *GH* and Prolactin (*Prl*) by E15.5 is the hallmark of the differentiation of somatotrope and lactotrope lineages respectively. Whilst gonadotropes are the last cell type to emerge, beginning at E16.5 with the onset of luteinizing hormone (LH) subunit β (*Lhb)* expression, followed by follicle-stimulating hormone (FSH) subunit β (*F* *shb) *a day later (Kelberman *et al*. 2009).

Although the classic description of cell differentiation is based on the sequential appearance of differentiating markers, recent birthdating studies imply that endocrine cells may be specified earlier and migrate some distance before they can be characterized by their differentiated markers. In fact, most of the hormone expressing cell types appear to differentiate between E11.5 and E13.5, denoting a broader range of specification rather than a sequential pattern of discrete times (Davis *et al*. 2011).

Concomitantly with these events, the hypothalamic primordium becomes morphologically evident in the neural ectoderm at E9.5 with neurogenesis commencing at E10, coinciding with the highest level of expression of genes important for the regional patterning of hypothalamic progenitor cells, such as *Sim1*, *Sim2*, *Arx* and *Nr5a1* (Shimogori *et al*. 2010). Hypothalamic neurogenesis is complete by E16 although expression of hypothalamic terminal differentiation markers peak postnatally (Shimogori *et al*. 2010). In the developing murine hypothalamus, transcription factors including *Gsh1, Mash1, Ash1, Sim1, Sim2, Arnt2, Brn-2,* and *Otp* are important for the differentiation of the parvocellular neurons secreting the neuropeptides TRH, thyrotropin-releasing hormone; GHRH, growth-hormone releasing hormone; SST, somatostatin; CRH, corticotropin-releasing hormone (Shimogori *et al*. 2010, Diaz *et al*. 2015). In the developing rat, hypothalamus Trh cells first appear at E10.5 in the ventral part of the anterior paraventricular area (VPa) and by E12.5 they are also found in the ventral portion of the paraventricular hypothalamic complex (TPVa) as well as in the central and dorsal portion of the peduncular paraventricular area (CPa and DPa) (Diaz *et al*. 2015). Recently, Trh expressing cells were also detected in the lateral hypothalamic area that is associated with behavioural response to motivation and metabolic stimuli (Horjales-Araujo *et al*. 2014).

### Genetic factors important for thyrotrope development

**Figure 2:**
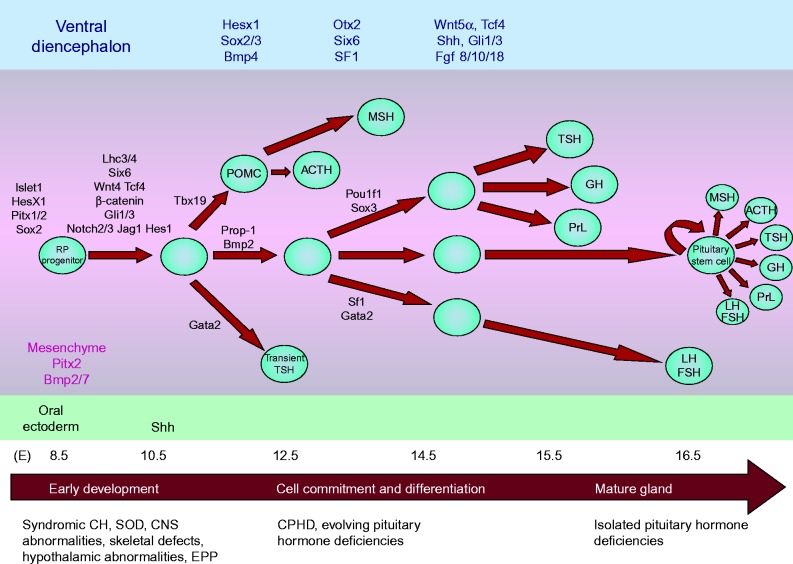
Schematic cascade of transcription factors and signaling molecule during pituitary development. Terminal differentiation of the anterior pituitary cell types is the result of complex interactions between extrinsic signalling molecules and transcription factors (*HESX1, SOX2, SOX3, OTX2, LHX3, LXH4, GATA2, IS* *L1, PROP1, POU1F1*). Possible pituitary phenotypes arising from mutations at different stages of pituitary development are indicated. SOD, septo-optic dysplasia; CNS, central nervous system; EPP, ectopic posterior pituitary; CPHD, combined pituitary hormone deficiency. Reproduced, with permission, from Kelberman D, Rizzoti K, Lovell-Badge R, Robinson IC, Dattani MT 2009 Genetic regulation of pituitary gland development in human and mouse, *Endocrine Reviews* 30 790–829. Copyright (2009) The Endocrine Society.

Signalling molecules from the ventral diencephalon (Bmp4, Fgf8, Fgf4, Nkx2.1, Wnt5α), the oral ectoderm (Sonic Hedgehog, Shh), the surrounding mesenchyme (Bmp2, Chordin) and the pouch itself (Bmp2, Wnt4) contribute to establish signalling gradients and the expression of transcription factors which will determine the positional identity of ventral pituitary cells (Zhu *et al*. 2007, Kelberman *et al*. 2009) (Fig. 2). Terminal differentiation of the anterior pituitary cell types is the result of complex interactions between extrinsic signalling molecules and transcription factors (*HESX1, SOX2, SOX3, OTX2, LHX3, LXH4, GATA2, ISL1*, *PROP1, POU1F1*) of which *GATA2, PITX1/2*, *PROP1* and *POU1F1* are most critical for the differentiation of thyrotropes.

Expression of Pitx1 is first detected in the anterior ectoderm at E8.0, then expressed throughout the oral ectoderm and in Rathke's pouch by E 9.5, and maintained throughout anterior pituitary development in all hormone-producing cell types. In the adult pituitary, Pitx1 expression is highest in thyrotropes expressing αGSU and in gonadotropes with lower levels in other hormone-producing cell types (Lanctot *et al*. 1999). Pitx1 null embryos have normal pituitary morphogenesis; however, at birth the number of thyrotropes and gonadotropes are reduced. This absence of early defects may in part be explained by the redundant function of the closely related PITX2.

Expression of PITX2 is detected widely in Rathke's pouch and in the developing pituirary, whilst in adults it is expressed predominantly in thyrotropes and gonadotropes. Mice with targeted deletion of Pitx2 in thyrotropes have smaller thyroid glands, upregulated levels of pituitary Pitx1 transcripts and circulating TSH and T_4_ in the normal range (Castinetti *et al*. 2011). In these animals, induction of hypothyroidism (by low iodine diet and oral propylthiouracil) results in a blunted TSH response. Although under these conditions control mice have significant increase in PITX1 transcripts, they remain unchanged in mutant animals (Castinetti *et al*. 2011).

Expression of Gata2 is first detected at E10.5 in the ventral Rathke's pouch, where it is induced by BMP2 along with αGSU, therefore marking the prospective and definitive thyrotropes and gonadotropes; its expression is maintained in the adult pituitary. *In vitro* GATA2 activates the *Cga* promoter and acts synergistically with POU1F1 to induce expression of *Tshb* (Gordon *et al*. 2002). Ectopic expression of Gata2 under the control of the Pou1f1 promoter results in dorsal expansion of the gonadotrope population at the expense of the Pou1f1 lineage (somatotropes, lactotropes and thyrotropes). Therefore, GATA2 may be required for the specification of gonadotropes and thyrotropes both in opposition and in synergy with POU1F1 (Dasen *et al*. 1999).

Knock-out of GATA2 specifically in the pituitary results in mice with fewer thyrotropes at birth, that exhibit growth delay postnatally and produce less TSH in response to severe hypothyroidism, compared to WT animals. The population of thyrotropes is only transiently reduced in neonates. However, in adult animals, levels of circulating TSH remain low and the function of thyrotropes is abnormal. These Gata2-deficient mice have increased levels of Gata3 transcripts in the pituitary gland, suggesting that the upregulation of GATA3 may have a compensatory role (Charles *et al*. 2006)

PROP1 (Prophet of PIT-1) is a pituitary-specific paired-like homeodomain transcription factor initially detected in the dorsal portion of Rathke's pouch at E10-10.5; its expression peaks at E12 and becomes undetectable by E15.5 dpc (Kelberman *et al*. 2009). The onset of Prop1 expression is required for the emergence of the Pou1f1 lineage (somatotropes, lactotropes and thyrotropes), whilst its persistent expression delays the differentiation of gonadotropes (Cushman *et al*. 2001). *In vitro* PROP1 and *β*-catenin form a complex, along with other cofactors that directly repress Hesx1 while activating expression of Pou1f1 (Olson *et al*. 2006)

The Ames dwarf mouse, that has a naturally occurring Prop1 mutation resulting in an eight-fold reduction in DNA-binding activity, has severe proportional dwarfism and infertility with GH, TSH and PRL deficiency, and reduced gonadotropin expression correlating with low plasma LH and FSH. The anterior pituitary gland in these animals is reduced in size by about 50% with an abnormal looping appearance (Ward *et al*. 2005, Ward *et al*. 2006).

Pou1f1 is expressed relatively late during pituitary development and is detectable in prospective somatotropes, lactotropes, and thyrotropes from E13.5. It reaches maximum expression in differentiating GH, PRL, and TSH cells by E16 and its extression persists in adulthood (Kelberman *et al*. 2009). POU1F1 is required for the production of GH, PRL, and TSHB as well as the expression of GHRHR. Two naturally occurring recessive mouse mutants, the Snell and Jackson dwarf mouse, exhibit an identical phenotype with postnatal, but not embryonic, anterior pituitary hypoplasia and GH, TSH, and PRL deficiencies (Kelberman *et al*. 2009). POU1F1 is able to inhibit GATA2, independently of its DNA binding properties, to prevent gonadotrope fate, whereas in thyrotropes, POU1F1 and GATA2 act synergistically to promote the thyrotrope fate (Dasen *et al*. 1999).

## Control of TSH biosynthesis

### The hypothalamic–pituitary–thyroid axis

#### Positive regulation of thyroid hormone synthesis

**Figure 3:**
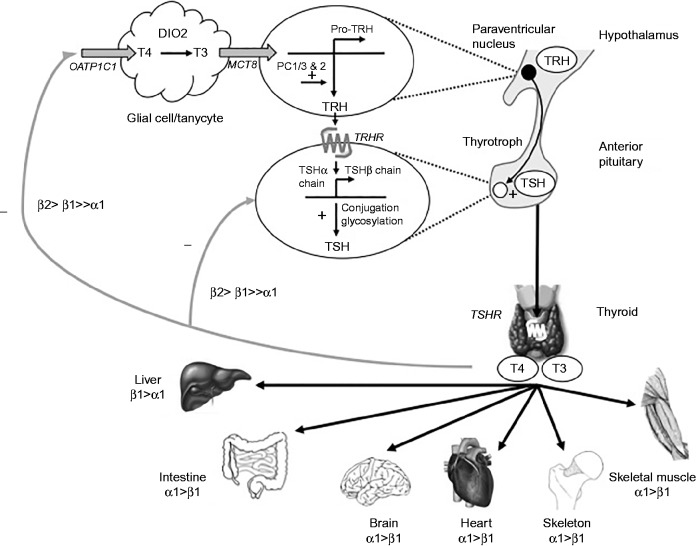
Diagramatic representation of the hypothalamic–pituitary–thyroid axis with positive regulation (black) predominantly mediated by thyrotropin-releasing hormone (TRH) and negative (grey) feedback influences, predominantly mediated by thyroid hormone receptor (TR) isoforms β2 and β1. Putative transporter molecules (grey) mediating these effects are annotated. OATP1C1 is expressed in capillaries throughout the brain, monocarboxyate transporter 8 (MCT8) is expressed in the PVN of the hypothalamus and in follicular stellate cells in the anterior pituitary (reviewed in Fliers *et al*. (2006)). Tissue-specific TR isoform expression is described, for thyroid hormone target tissues.

Circulating concentrations of T_3_ and T_4_ are maintained within a narrow range *in vivo* by a highly regulated balance of positive and negative feedback mediated by the hypothalamic–pituitary–thyroid (HPT) axis and centrally regulated by TRH (Fig. 3). TRH is synthesized as a prohormone in the paraventricular nucleus (PVN) of the hypothalamus and matures into the TRH tripeptide amide (pGlu-His-ProNH2) following post-translational cleavage by prohormone convertases PC1/3 and 2. After axonal transport to the median eminence, TRH reaches the thyrotrophs of the anterior pituitary gland via the hypothalamic portal vein, where it binds its G-protein coupled receptor, TRHR, and activates a Gq/11 dependent pathway involving mobilization of intracellular calcium and activation of protein kinase C, culminating in TSH synthesis and secretion. Pyroglutamyl peptidase II (PPII) activity subsequently mediates degradation of extracellular TRH (Hinkle *et al*. 2012, Fekete & Lechan 2014). Recent data suggests a role for the membrane glycoprotein IGSF1 in normal TRHR expression, although the precise function of IGSF1 remains unclear (Sun *et al*. 2012).

Besides upregulating transcription of the TSH alpha (αGSU) and β subunit genes (*CGA* and *TSHB*), TRH mediates conjugation of TSH α and β subunits and regulates glycosylation and secretion of heterodimeric TSH. Incorporation of oligosaccharide determines TSH bioactivity, which is principally defined by the type and conformation of carbohydrate chain added at Asn-76 and -102 of the α subunit and Asn-43 on the β subunit such that TSH with a high sialic acid content exhibits decreased bioactivity and increased half life. (Persani 1988, Estrada *et al*. 2014).

In the thyroid, TSH binds a G-protein coupled receptor, TSH-receptor (TSHR), stimulating follicular cell growth and thyroid hormone synthesis and release, predominantly in the form of the prohormone thyroxine (T_4_). Deiodination of T_4_ in peripheral tissues yields the active hormone, 3,5,3′-triodothyronine (T_3_) which binds thyroid hormone receptors (TR's) to exert its transcriptional effects. TR isoforms TRα1, β1, and β2 exhibit tissue-specific expression patterns and heterodimerize with retinoid X receptor (RXR) on specific Thyroid Receptor DNA response elements (TREs), usually located in promoter regions of target genes. With upregulated genes, unliganded TRs mediate repression of basal gene transcription by binding the TRE together with other proteins, eg corepressors (NCOR1 and SMRT) and histone deacetylases (HDAC3) (Cheng *et al*. 2010). T_3_ binding results in a conformational change of the receptor ligand-binding domain, leading to coactivator recruitment, histone acetylation, relaxation of chromatin and transcriptional activation (Ortiga-Carvalho *et al*. 2014).

#### Negative regulation of thyroid hormone synthesis

A classical negative feedback loop maintains circulating thyroid hormone levels within the normal range and is predominantly mediated by TRβ2 (Fig. 3). In the hypothalamus, thyroid hormone is taken up into the brain from cerebrospinal fluid (CSF) in the 3rd ventricle or from blood vessels in the median eminence by deiodinase type 2 (DIO2)-expressing tanycytes or astrocytes respectively. DIO2, converts T_4_ to T_3_ which then enters the TRH neurons and binds nuclear TR's. Intracellular delivery of thyroid hormones requires active transport, likely to be mediated by monocarboxylate transporter eight (MCT8) for uptake from tanycytes into neuronal cells and by organic anion-transporting polypeptide 1c1 (OATP1C1) for transport across the blood-brain barrier (Alkemade *et al*. 2011, Garcia *et al*. 2014, Alkemade 2015). A shorter T_3_-mediated negative feedback loop operates in the anterior pituitary, with the transport mechanism for pituitary thyroid hormone uptake remaining poorly defined. TSHR expression has also been demonstrated in pituitary folliculostellate cells, leading to the suggestion that paracrine signaling in the pituitary may also contribute to negative feedback, but the mechanisms underlying this remain speculative (Prummel *et al*. 2004, Garcia *et al*. 2014).

In the hypothalamus, thyroid hormones downregulate transcription of the pro-TRH and PC1/3 and PC2 genes (Hollenberg *et al*. 1995, Perello *et al*. 2006) resulting in reduced levels of mature TRH. In the anterior pituitary, transcription of *CGA* and *TSHB* genes is inhibited (Shupnik *et al*. 1985, Wondisford *et al*. 1989, Wang *et al*. 2009). The mechanisms for negative regulation of transcription by TRs remain poorly understood (Ortiga-Carvalho *et al*. 2014) but may involve recruitment of corepressors instead of coactivators, eg Ncor1 during transcriptional repression of murine *Cga*, (You *et al*. 2010) *Trans*-repression, where liganded TR's interact with and inhibit the activity of other transcription factors, has also been implicated, eg GATA2 during TR-mediated repression of *TSHB* (Matsushita *et al*. 2007, Santos *et al*. 2011).

Additional modulators of TSH secretion include hypothalamic dopamine and somatostatin (inhibitory), and the influence of feeding behaviour, glucocorticoids, severe illness, cold, and circadian rhythm (Fliers *et al*. 2006).

## Genetic causes of central congenital hypothyroidism

Isolated central hypothyroidism is a rare entity, with an estimated incidence of 1:65 000 and known genetic causes affect the TSH biosynthetic pathway, comprising mutations in *TSHB* and *TRHR*, and *IGSF1 *(Joustra *et al*. 2013*a*). In the majority of patients, CCH occurs in the context of combined pituitary hormone deficiencies, and additional syndromic features may manifest depending on its genetic aetiology. Only a minority of individuals will harbour mutations in known transcription factors such as *POU1F1*, *PROP1*, *HESX1*, *LHX3*, *LHX4*, *SOX3* and *OTX2* that are implicated in pituitary development (Alatzoglou & Dattani 2009, Kelberman *et al*. 2009). This suggests that other, as yet unidentified genetic or epigenetic factors, may be implicated in the aetiology of CCH.

## Isolated central congenital hypothyroidism

### *TSHB* mutations

Biallelic loss of function, *TSHB *mutations are associated with severe central hypothyroidism of neonatal onset for which additional biochemical hallmarks include elevated pituitary glycoprotein α subunit, and an impaired TSH response to TRH administration, despite a preserved rise in serum Prolactin (Bonomi *et al*. 2001) (Table 1). Concomitant neurodevelopmental impairment is a frequent finding, and usually correlates with treatment delay due to the fact that individuals evade detection on TSH-based, primary CH screening programmes, and remain undiagnosed until their profound congenital hypothyroidism manifests clinically. In cases who are diagnosed and treated from birth due to ascertainment following a prior genetic diagnosis in their family, developmental outcome is often improved (Brumm *et al*. 2002, Karges *et al*. 2004).

**Figure 4:**
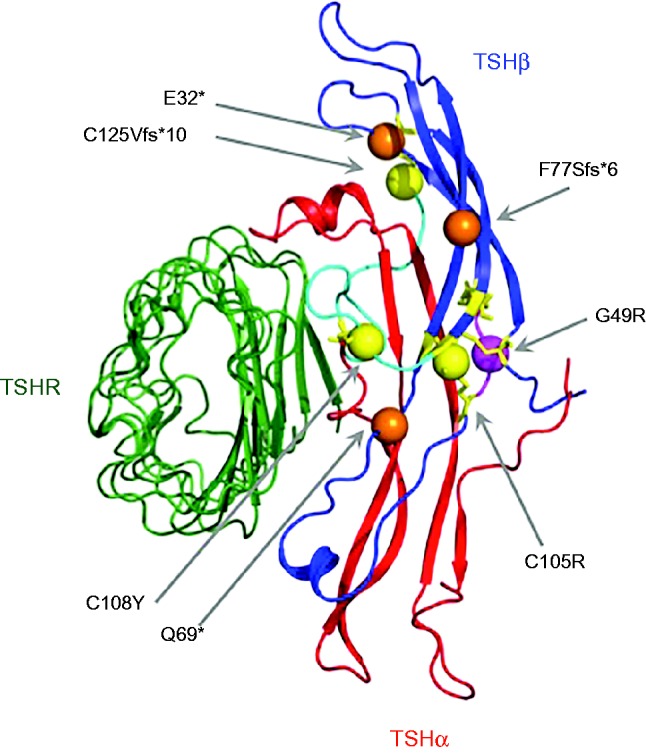
Model for heterodimeric thyroid stimulating hormone (TSH) bound to the TSH receptor (TSHR) illustrating the position of naturally occurring TSHB loss-of-function mutations associated with congenital central hypothyroidism. The model was generated using PHYRE for predicting TSHbeta subunit (TSHb) structure and was modelled onto FSH-FSHR (1×wd) and the TSHR-K1-70FAB (2×wt) structure using PYMOL. Colour coding is as follows: Green TSHR, Red: TSH alpha subunit (TSHa, aGSU), Blue: TSHb. Specific structural features required to maintain the heterodimeric structure: Cyan ‘seatbelt’ region, Yellow: cysteines conserved throughout cysteine knot proteins and involved in disulphide bridge formation. Spheres denote TSHB mutations: C105R; C108Y; C125Vfs*10 (yellow) disrupt disulphide bridges, G49R (purple) is located in the conserved CAGYC region and E32*; Q69*; F77Sfs*6 (orange) truncate the protein prematurely. The nomenclature of these mutations follows the most recent HGNC guidelines to include the 20 amino acid signal peptide of TSHB, thus may differ from that cited in the original articles. Nomenclature can be converted to that previously published for missense mutations by subtracting 20 eg Q69* new nomenclature=Q49X old nomenclature.

Heterodimeric TSH comprises the common α subunit (αGSU) shared with other glycoprotein hormone (LH, FSH, CG) family members and a hormone-specific β-subunit (TSHB) in which naturally-occurring mutations either truncate the protein, or perturb key structural features required for heterodimeric integrity (Fig. 4). In common with other cysteine-knot proteins, a central cysteine knot in each subunit is surrounded by two β-hairpin loops on one side, and a long loop on the other. A ‘seat belt’ formed from the TSH β subunit, wraps around the long loop of the α-subunit and forms an intra-molecular disulfide ‘buckle’ to stabilize the heterodimer and additional α-β subunit interactions occur around a conserved CAGYC sequence motif (Szkudlinski *et al*. 2002, Jiang *et al*. 2014). Conserved cysteine residues in all glycoprotein hormone β-subunits form disulphide bridges central to the three-dimensional structure of the protein, and alignment with β-hCG, for which the crystal structure has been solved, predicts similar interactions in TSHB. Disulphide bridges between Cys22-Cys72, Cys47-Cys103, and Cys51-Cys105 are thought to maintain the cystine-knot motif; Cys39-Cys125 and Cys108-Cys115 are thought to be integral to ‘seatbelt’ formation; and an interaction between Cys37-Cys87 is thought to link the two β-hairpin loops (Szkudlinski *et al*. 2002, Jiang *et al*. 2014).

Nine, naturally-occurring, *TSHB* mutations have been described, including missense (C108Y, C105R, G49R), nonsense and frameshift mutations (p.E32*, p.Q69*, p.C125Vfs*10, p.F77Sfs*6) (Hayashizaki *et al*. 1989, Dacou-Voutetakis *et al*. 1990, Medeiros-Neto *et al*. 1996, Bonomi *et al*. 2001, Vuissoz *et al*. 2001, Sertedaki *et al*. 2002, Morales *et al*. 2004, Baquedano *et al*. 2010), as well as two splice-site mutations (c162G>A, c.162+5 G>A) (Pohlenz *et al*. 2002, Baquedano *et al*. 2010). More recently, a homozygous *TSHB* deletion was reported (Hermanns *et al*. 2014). All the missense mutations disrupt key disulphide bridges required for heterodimeric integrity or disrupt the CAGYC region (Fig. 4). (For the purposes of this review, the nomenclature of these mutations follows the most recent HGNC guidelines to include the 20 amino acid signal peptide of TSHB, such that the annotation may differ from that cited in the original published articles.)

The most frequently described mutation is a single nucleotide deletion (c373delT) leading to a cysteine 125 to valine change (p.C125V) and subsequent frameshift and premature stop codon at position 134 (p.C125Vfs*10) (Medeiros-Neto *et al*. 1996). This has been reported worldwide in several, non-consanguineous families, although a founder effect was also described in three German kindreds (McDermott *et al*. 2002, Brumm *et al*. 2002, Deladoey *et al*. 2003, Domene *et al*. 2004, Karges *et al*. 2004). Functional studies have demonstrated that replacement of the cysteine at position 125 with valine, rather than subsequent deletion of the terminal 13 amino acids, impairs the bioactivity of the mutant TSH, by disrupting the Cys125-Cys39 disulphide bond which forms the ‘buckle’ of the TSHB ‘seatbelt’ surrounding the αlpha subunit (Medeiros-Neto *et al*. 1996).

Mutations (p.G49R, p.Q32*), which disrupt heterodimer formation between TSHα and β polypeptides generally result in unmeasurable serum TSH concentrations, whereas mutations (eg p.Q69*, IVS2+5 G>A, c.373delT), which preserve formation of some mutant, heterodimeric TSH expressing the epitopes recognized by the anti-TSH monoclonal antibody, enables TSH to be detected in an immunoassay-dependent manner. Thus, some cases may have detectable levels of immunoreactive TSH, but these species will lack normal bioactivity (Bonomi *et al*. 2001). In contrast, a recent report describes a TSHB variant with impaired immunoreactivity but normal bioactivity (c.223A>G, p.R75G including the signal peptide). Two clinically euthyroid siblings, both homozygous for the variant, exhibited normal thyroid hormone levels but undetectable TSH specifically in Siemens assay platforms, due to poor TSH adsorption by the monoclonal antibody (Pappa *et al*. 2015). Although affected individuals are euthyroid, this variant had previously been detected in South East Asian patients, some of whom had consequently been inappropriately treated with antithyroid drugs (Drees *et al*. 2014). Alternatively, the undetectable TSH may erroneously suggest central hypothyroidism on CH screening.

### *TRHR *mutations

Biallelic *TRHR* mutations are the least common cause of isolated congenital hypothyroidism, and have hitherto been described in only three cases from two unrelated kindreds. In both families, the male Probands exhibited subnormal T_4_ concentrations 40% to 88% lower limit of the normal range, with associated clinical manifestations predominantly comprising growth retardation and a delayed bone age. There was reportedly no attributable neurological deficit despite presentation of patients at age 9 and 11 years, suggesting preservation of sufficient thyroid hormone production in infancy to prevent overt mental retardation. Pituitary TSH synthesis was inadequate (in conjunction with low T_4_, serum TSH was inappropriately normal), but circulating T_4_ levels rose appropriately following levothyroxine withdrawal, indicating that synthesis of bioactive TSH could occur in the absence of TRH signalling (Collu *et al*. 1997, Bonomi *et al*. 2009). Rhythmic TSH secretion was also preserved and pituitary morphology was normal (Collu *et al*. 1997, Bonomi *et al*. 2009).

Intriguingly, a female with a homozygous, nonsense *TRHR* mutation (p.R17*), was only diagnosed with central hypothyroidism following family screening at the age of 33, having previously achieved two normal pregnancies with subsequent lactation. Although TRHR is expressed in lactotrophs as well as thyrotrophs, and stimulates prolactin secretion in response to i.v. TRH administration, its physiological role in these cells is unclear. Both male and female individuals with biallelic TRHR mutations had absent TSH and prolactin responses to TRH administration. However, the obstetric history in the p.R17* homozygous female suggests that TRH action is not obligatory for pregnancy and lactation in humans (Bonomi *et al*. 2009).

**Figure 5:**
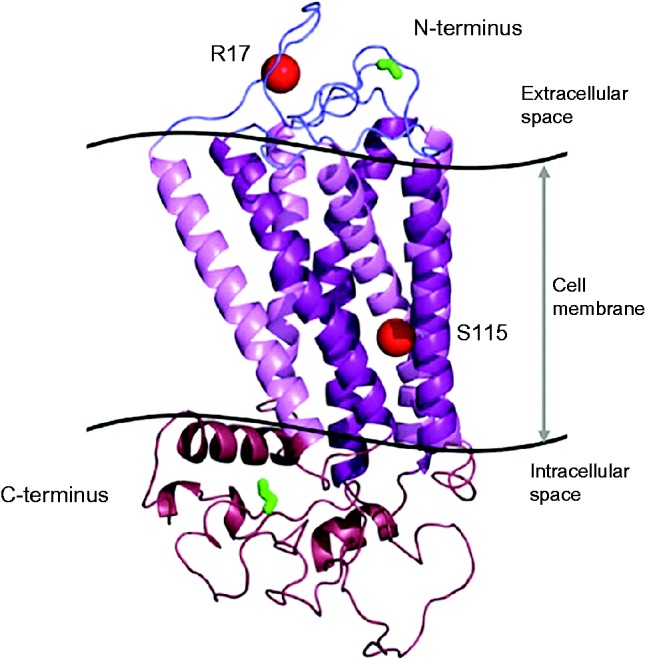
Crystallographic modeling of TRHR showing the positions (red spheres) of the two previously described mutations associated with central hypothyroidism: R17X truncating the protein in the extracaellular domain and an in-frame deletion of 3 amino acids (Ser115-Thr117) plus a missense change (Ala118 for Thr118; p.S115-T117del+T118) located at the cytoplasmic end of the third transmembrane domain of the receptor The TRHR structural model was generated by homology modeling using the PHYRE server and Pymol. The N-terminal start codon and C-terminal end codon are highlighted in green.

In common with all family A G-protein-coupled receptors (GPCR's), the structure of TRHR includes an extracellular N-terminus and intracellular C-terminus, flanking seven transmembrane domains connected by three intracellular and three extracellular loops, which form a ligand-binding pocket within the plasma membrane. TRH-binding results in a conformational change involving transmembrane helices 5 and 6 and the third intracellular loop, with subsequent carboxyterminal domain-mediated G-protein coupling (Hinkle *et al*. 2012) (Fig. 5). In both families with TRHR defects, the mutations have been highly deleterious, resulting in complete abrogation of TRHR function. The first case was compound heterozygous for a maternally-inherited nonsense mutation (p.R17*) truncating the protein before the transmembrane domains, and a paternally-inherited in-frame deletion of three amino acids (S115, I116, and T117) with one substitution (p.A118T) in the third transmembrane domain. In the second reported kindred, affected individuals were homozygous for the p.R17* mutation (Collu *et al*. 1997, Bonomi *et al*. 2009) (Fig. 4).

Endocrine abnormalities in TRHR null humans are recapitulated in mice lacking *Trhr1* who also exhibit subnormal T_4_ and T_3_ levels with inappropriately normal TSH (Table 1). Normal somatotrope, thyrotrope, and lactotrope numbers confirm that TRHR is not needed for the development or maintenance of TRH target cells, with preservation of fertility, pregnancy and lactation in females although both basal serum prolactin and lactation-stimulated prolactin mRNA are decreased. Increased fasting glucose concentrations were also observed in the mice. However, there is currently no evidence for an extrapituitary phenotype in humans with *TRHR* mutations (Rabeler *et al*. 2004, Zeng *et al*. 2007).

### *IGSF1* mutations

Mutations in the immunoglobulin superfamily member 1 (*IGSF1*) gene are the most recently identified cause of central hypothyroidism, with an estimated incidence of up to 1:100 000 (Sun *et al*. 2012, Joustra *et al*. 2013*a*). The X chromosome located *IGSF1* gene encodes a membrane glycoprotein which was first thought to be an inhibin co-receptor in the pituitary, but recent binding and *in vivo* data from mice and humans suggests that this is unlikely (Chong *et al*. 2000, Chapman & Woodruff 2001, Chapman *et al*. 2002, Bernard *et al*. 2003). At mRNA level, IGSF1 and its murine homolog Igsf1 are abundantly expressed in Rathke's pouch (the developing pituitary primordium) and in adult pituitary gland (Sun *et al*. 2012). A paucity of reliable antibodies has hampered expression studies of the human protein; however, in murine pituitary, IGSF1 protein is detected in thyrotropes, somatotropes and lactotropes, but not gonadotropes, and in the rat, pituitary expression is also confined to cells of the Pou1f1 lineage (Sun *et al*. 2012, Joustra *et al*. 2015*a*). Although a role for IGSF1 in pituitary and hypothalamic physiology is supported both by its cellular expression pattern and by endocrine consequences of IGSF1 deficiency in mice and humans, its precise physiological function in both species remains undefined (Sun *et al*. 2012).

**Figure 6:**
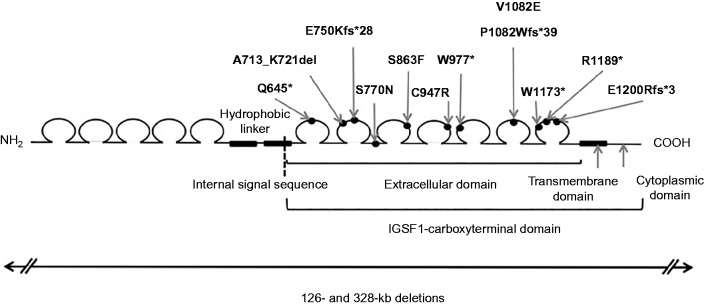
Schematic illustrating the protein domain structure of IGSF1 with the internal signal peptide directing cleavage of the carboxy-terminal domain denoted by a dashed line. Positions of naturally-occurring mutations associated with congenital central hypothyroidism are denoted; all are located within the carboxyterminal domain. Two whole gene deletions (below) have also been reported (Sun *et al*. 2012, Nakamura *et al*. 2013, Tajima *et al*. 2013).

The IGSF1 protein undergoes co-translational proteolysis such that the carboxyterminal portion trafficks to the plasma membrane where it is expressed as a large extracellular domain with a short intracellular cytoplasmic tail (Robakis *et al*. 2008). Fourteen pathogenic mutations in the IGSF1 gene have been described, in cases from the Netherlands, the UK, Italy and Japan (Sun *et al*. 2012, Nakamura *et al*. 2013, Tajima *et al*. 2013), all of which impair either protein maturation or membrane trafficking, resulting in decreased plasma membrane expression of IGSF1. The twelve missense or truncating mutations are all located in the extracellular portion of the carboxyterminal domain; the remaining two mutations are whole gene deletions (Fig. 6).

Four publications have documented the consequences of loss of function mutations in IGSF1 in humans (Sun *et al*. 2012, Joustra *et al*. 2013b, Nakamura *et al*. 2013, Tajima *et al*. 2013). All affected males exhibited central hypothyroidism, either isolated, or associated with hypoprolactinaemia (subnormal basal prolactin levels) (67% cases). A minority of patients required treatment for transient, partial, growth hormone deficiency in childhood; paradoxically, in some European cases, circulating IGF1 levels tended to increase with age relative to the age-matched reference interval and several patients developed acromegaloid features in late adulthood. Pubertal development was usually disharmonious, with a delayed pubertal growth spurt and testosterone rise despite normal onset of testicular growth. All evaluable European males subsequently developed adult macroorchidism, with ultrasonographic testicular volumes close to 50 ml in two cases (Sun *et al*. 2012, Joustra *et al*. 2013b). Additionally, phenotyping of female *IGSF1* mutation carriers indicated that although IGSF1 deficiency is X-linked, one-third of heterozygous females exhibit central hypothyroidism, and up to 11% demonstrate hypoprolactinaemia. Intriguingly, four of 18 females investigated had undergone surgical resection of benign ovarian cysts, suggesting possible shared pathogenetic mechanisms with the macroorchidism observed in males (Table 1). Skewed X inactivation was not a reliable predictor of endocrinopathy in female cases (Joustra *et al*. 2013b).

Murine studies have suggested that impaired TRH signaling may play a role in the central hypothyroidism associated with IGSF1 deficiency. Male igsf1 deficient mice are centrally hypothyroid, with decreased TRHR mRNA expression in the pituitary. In keeping with impaired TRH signaling, serum T_3_ and TSH levels are subnormal despite normal pituitary TSHB mRNA synthesis; and there is a blunted TSH response to TRH administration. Hypothalamic TRH mRNA levels are elevated, suggesting intact synthesis of TRH (Bernard *et al*. 2003, Sun *et al*. 2012) (Table 1). Most humans with hemizygous IGSF1 mutations exhibit biochemical hypothyroidism that is comparable to biallelic TRHR mutation cases, comprising mild-moderate CCH with detectable TSH and apparently normal neurological development even when the diagnosis is delayed until adulthood. These features would be consistent with impaired TRH signaling being the basis of the central hypothyroidism phenotype in the human IGSF1 deficiency, with such defective signaling also predicted to result in decreased bioactivity of TSH. Such qualitative assessment of TSH, either directly or indirectly by measurement of rise in circulating T_3_ and T_4_ increment during a prolonged TRH stimulation test, would therefore be informative in this context (Yamada & Mori 2008).

The mechanisms underlying the macroorchidism and disharmonious pubertal development seen in IGSF1 deficient males remain to be ascertained, and mechanistic possibilities remain speculative. Excess hypothalamic TRH, acting on gonadotropes which express TRHR but not IGSF1, could drive macroorchidism via an FSH-mediated process, consistent with observations that serum FSH was always higher than serum LH, in IGSF1-deficient males (Kugler & Huseman 1983, Ryan *et al*. 2007, Sun *et al*. 2012). Alternative contributory factors could include hypothyroxinaemia acting directly to increase Sertoli cell number and potentially also underlying the delayed testosterone rise (Weber *et al*. 2003, Holsberger & Cooke 2005, Joustra *et al*. 2013*a*), although the absence of macroorchidism in other forms of congenital central hypothyroidism argues against this as the sole cause. A further possibility would be a direct testicular effect of IGSF1, but it remains unclear whether IGSF1 protein is expressed in human testis. Rats exhibit testicular expression of IGSF1 in adult Sertoli cells during stages XIII through VI of the seminiferous epithelium, and in elongating spermatids during epithelial stages X through XIII (Joustra *et al*. 2015*a*). IGSF1 deficient mice exhibit greater testicular weight but this is proportional to their increased body mass, and they do not express IGSF1 protein in testis (Sun *et al*. 2012).

Hitherto, studies of the endocrine effects of IGSF1 deficiency may been confounded by selection bias, with gene screening being undertaken predominantly in cases of central hypothyroidism. More recently, *IGSF1* has been sequenced in euthyroid patients with X-linked constitutional delay of growth and puberty, and individuals with gigantism or acromegaly, with no evidence for a primary causative role for genetic variation in *IGSF1* in these disorders (Faucz *et al*. 2015, Joustra *et al*. 2015*b*).

IGSF1 remains a highly polymorphic gene with an as yet incompletely characterized role both in anterior pituitary cells of the POU1F1 lineage, and in pubertal and gonadal development. Additionally, significant phenotypic variability amongst individuals harbouring IGSF1 mutations, suggests that its role may be influenced by as yet unidentified genetic or environmental modifiers.

## Combined pituitary hormone deficiencies

Central congenital hypothyroidism in association with combined pituitary hormone deficiencies may be i) syndromic, resulting from mutations in early transcription factors, and associated with developmental abnormalities (eg septo-optic dysplasia and its variants, holoprosencephaly and midline defects, ocular or skeletal defects, and intellectual impairment) or ii) non syndromic resulting from mutations in late transcription factors (*PROP1, POU1F1*). The variable phenotypes, associations and MRI findings are summarized in Table 1.

Septo-optic dysplasia (SOD) is defined by the combination of two of the following i) optic nerve hypoplasia (ONH) ii) midline forebrain defects (ie agenesis of the corpus callosum, absent septum pellucidum) and iii) pituitary hypoplasia with variable hypopituitarism. The commonest endocrine defect is GH deficiency followed by TSH and ACTH deficiency, whilst gonadotropin secretion may be retained. Mutations in *HESX1, OTX2, *and *SOX3* have been identified in patients with TSH deficiency and SOD (Dattani *et al*. 1999, Woods *et al*. 2005, McNay *et al*. 2007). There is increasing evidence of overlap in the aetiology of conditions that were previously considered to be discrete, such as Kallmann syndrome, SOD and combined pituitary hormone deficiencies as mutations in the same array of genes (*KAL-1, PROKR2, FGF8, FGFR1*) have now been implicated in their aetiology. In this respect, the TSH deficiency may be part of the initial presentation or evolving phenotype of these patients (McCabe *et al*. 2011, McCabe *et al*. 2013).

Mutations in the Lim homeodomain transcription factors (*LHX3*, *LHX4*) lead to multiple pituitary hormone deficiencies, including TSH deficiency. Expression of *Lhx3* is initially detected uniformly within Rathke's pouch from E9.5 and by E16.5 it is expressed in the developing anterior and intermediate pituitary where its expression persists into adulthood. Mice with a targeted homozygous disruption of *Lhx3* die shortly after birth and exhibit pituitary aplasia. Although Rathke's pouch is initially formed, its expansion is arrested by E12.5 and lacks almost all of the hormone-secreting cell types, containing only a small population of corticotropes (Mullen *et al*. 2007, Kelberman *et al*. 2009). Patients with homozygous or compound heterozygous *LHX3* mutations have an evolving endocrine phenotype involving GH, PRL, LH, FSH and TSH deficiency (Mullen *et al*. 2007). Although ACTH seems to be spared in the majority of patients, ACTH deficiency has also been described (Rajab *et al*. 2008). The anterior pituitary morphology is variable (hypoplastic, enlarged or presence of microadenoma) with a eutopic posterior pituitary. Patients may have cervical abnormalities, with or without restricted neck rotation, sensorineural hearing loss (Netchine *et al*. 2000, Mullen *et al*. 2007).

*Lhx4* is closely related to *Lhx3; *it is initially expressed throughout the Rathke's pouch at E9.5. However, in contrast to *Lhx3*, its expression is transient, restricted to the future anterior lobe and down-regulated by E12.5. Homozygous Lhx4 null mice die shortly after birth from lung defects; the anterior lobe of Lhx4^−/−^ mice contains all of differentiated cell types but there is reduction in cell proliferation (Mullen *et al*. 2007, Kelberman *et al*. 2009). Patients with heterozygous missense or frameshift *LH* *X4* mutations have GH and variable gonadotrophin, TSH and ACTH deficiencies, a hypoplastic anterior pituitary with/without an undescended/ectopic posterior pituitary and other abnormalities including a poorly formed sella turcica, pointed cerebellar tonsils or Chiari malformation (Castinetti *et al*. 2008, Pfaeffle *et al*. 2008)

Mutations in the transcriptional repressor HESX1 are associated with SOD, CPHD and IGHD (Dattani *et al*. 1998, Carvalho *et al*. 2003, McNay *et al*. 2007, Kelberman *et al*. 2009). Endocrine deficits may include GH, TSH, ACTH and gonadotrophin deficiencies. The anterior pituitary may be hypoplastic or absent whereas the posterior pituitary is usually ectopic but occasionally may be eutopic. In mice, *Hesx1* is expressed in the anterior neural ectoderm, and thereafter in the forebrain and developing pituitary, although its expression is switched off in the forebrain at E9, and in Rathke's pouch after E13.5. Homozygous null mutant mice have severe eye, forebrain and pituitary defects (Dattani *et al*. 1998, Sajedi *et al*. 2008), a phenotype that is reminiscent of SOD.

OTX2 (Orthodentic homeobox 2) is a transcription factor required for the formation of anterior structures and maintenance of the forebrain and has been implicated in 2–3% of anophthalmia/microphthalmia syndromes in humans (Boncinelli & Morgan 2001, Kurokawa *et al*. 2004). In mice, the expression of Otx2 is localised to developing brain, eye, nose and ear. Homozygous knockout mice die at midgestation with severe brain abnormalities, whereas heterozygous mutants have a variable phenotype ranging from normal to severe eye and brain abnormalities (anophthalmia, holoprosencephaly or anencephaly) (Acampora *et al*. 1995). Heterozygous mutations in *OTX2* have highly variable pituitary phenotypes that range from partial IGHD to hypopituitarism, with or without an ectopic posterior pituitary and, rarely, even without an ocular phenotype (Dateki *et al*. 2008, Diaczok *et al*. 2008, Tajima *et al*. 2009).

Over- and under-dosage of *SOX3* has been implicated in the aetiology of X-linked hypopituitarism with a highly variable phenotype ranging from isolated growth hormone deficiency to combined pituitary hormone deficiency, including evolving TSH deficiency, with or without variable mental retardation or learning difficulties. (Laumonnier *et al*. 2002, Woods *et al*. 2005, Alatzoglou & Dattani 2009). MRI findings include an undescended posterior pituitary, anterior pituitary hypoplasia, or persistence of the craniopharyngeal canal (Woods *et al*. 2005, Alatzoglou *et al*. 2014).

*PROP1* mutations are the most common cause of CPHD, including GH, TSH, gonadotropin, and evolving ACTH deficiencies of variable onset. Recessive *PROP1* mutations are associated with GH, TSH, prolactin and gonadotropin deficiency, although the timing and extent of these deficits vary and the full phenotype may not be evident from the outset. For instance, patients homozygous for the p.R120C mutation may first present in childhood with GHD before the later development of TSH, prolactin and gonadotropin deficiencies (Fluck *et al*. 1998). Most patients with *PROP1* mutations have a hypoplastic or normal anterior pituitary gland with a eutopic posterior pituitary. However, there have been reports of an enlarged anterior pituitary at initial scanning in childhood with spontaneous involution over time, often waxing and waning before eventual involution (Voutetakis *et al*. 2004, Turton *et al*. 2005).

Patients with autosomal recessive and dominant *POU1F1* mutations have GH and PRL deficiencies, that are generally present from early life, and are associated with a normal or hypoplastic anterior pituitary, whilst TSH deficiency can be highly variable. Although the majority have early TSH deficiency, hypothyroidism may also occur later in childhood. A patient with a *POU1F1* mutation identical to that found in an unrelated patient who developed central hypothyroidism in the second year of life, has been reported with GH and PRL deficiency and yet normal thyroid function at the age of 21 years. (Turton *et al*. 2005).

## Diagnosis of central congenital hypothroidism

The TSH-based protocol used by most neonatal CH screening programmes will unfortunately only detect primary CH since CCH is usually associated with inappropriately normal or low TSH. However, the merits of screening for CCH remain a subject of debate with arguments against screening citing its relative rarity and the presumption that it is usually mild and not likely to be associated with brain damage (Price & Weetman 2001, La Franchi 2010, La Franchi 2011). In the Netherlands, an unique screening algorithm based on combined measurement of TSH, T_4_ and thyroxine binding globulin (TBG) results in a diagnosis of CCH in as many as 1 in 16 000 newborns, the majority of whom will have additional pituitary hormone deficits (van Tijn *et al*. 2005, Kempers *et al*. 2006). Advantages to case detection by screening include prevention of life-threatening hypoglycaemia due to coexisting GH and/or ACTH deficiency in these individuals as well as enabling early neonatal levothyroxine replacement. A recent study demonstrated that more than 50% of children with CCH will have moderate or severe hypothyroidism, such that the potential neurological sequelae of delayed diagnosis should not be underestimated (Persani 2012, Zwaveling-Soonawala *et al*. 2015). However, although the recent ESPE CH guidelines acknowledge that screening for CCH fulfils generally accepted disease screening criteria, studies confirming superiority of detection through screening compared with case identification by clinical ascertainment are lacking (Fisher 2005, Leger *et al*. 2014).

A high index of suspicion is required when investigating for CCH, since early clinical diagnosis is often difficult, and unless the hormone deficit is profound, classical signs of hypothyroidism may be absent. The mainstay of biochemical diagnosis remains the association of low free T_4_ concentrations with inappropriately low or normal TSH concentrations in the absence of immunoassay interference (Ferretti *et al*. 1999, Beck-Peccoz 2011, Persani 2012). Circulating T_3_ levels are often normal, due to increased DIO2 activity (Alexopoulou *et al*. 2004, Sun *et al*. 2012).

Although biochemical diagnosis of overt central hypothyroidism is generally unequivocal, detection may be more challenging in mild cases where fT_4_ is only marginally subnormal or when hypothalamic dysfunction results in elevated immunoreative TSH with subnormal bioactivity (Beck-Peccoz 2011). In situations where central thyroid dysfunction evolves with time (eg in children with *POU* *1F1* mutations), declining fT_4_ concentrations may be an early indicator of CCH, with a time-related decline in fT_4_ of >20% quoted diagnostically in individuals with acquired pituitary disease (Alexopouou *et al*. 2004). Biochemical markers of thyroid hormone action (eg CPK, cholesterol), lack diagnostic sensitivity but may have a role in monitoring treated cases and may support a diagnosis of CCH (Ferretti *et al*. 1999). A recent study suggests that T_3_-dependent echocardiographic parameters may be useful in identifying adults with subclinical central hypothyroidism (Doin *et al*. 2012). Additional tests that may have a diagnostic role in mild CCH include evaluation of the nocturnal TSH surge, which is usually absent or blunted even in subclinical cases (Rose *et al*. 1990, Rose 1995, Roelfsema & Veldhuis 2013). The TSH index, in which a log-linear relationship between fT_4_ and TSH is used to predict the normal amount of feedback-induced change in log TSH per change in fT_4_, has also been proposed as an estimate of pituitary thyrotroph function, although extrapolation of these results to the paediatric population is challenging (Jostel *et al*. 2009).

The role of TRH testing in CCH remains controversial, both at diagnosis and in discriminating between pituitary and hypothalamic pathology. Initial studies found a blunted TSH rise to be indicative of pituitary hypothyroidism, whereas an exaggerated, delayed or prolonged TSH response was associated with tertiary hypothyroidism (Costom *et al*. 1971, Faglia *et al*. 1973). However, more recent studies in children with central hypothyroidism suggest that TRH testing has both a poor negative predictive value, and an inability to distinguish between hypothalamic and pituitary defects (Mehta *et al*. 2003). In a study of 54 children with central hypothyroidism, 23.3% had a normal TRH test, only 30% had an absent or blunted TSH response suggestive of pituitary disease, whilst 30% had a delayed hypothalamic response and 16.7% a brisk response (Mehta *et al*. 2003). In a different setting, a prospective study of twenty infants diagnosed with central hypothyroidism on neonatal screening, concluded that TRH testing is useful for diagnosis and for distinguishing between patients likely to have isolated or combined pituitary deficiencies, provided that the TSH response to TRH is assayed for at least 180 min post hormone administration. In this study, the majority of patients with type 2 responses had isolated TSH deficiency (67%), whilst all patients with type 3 TSH responses to TRH had combined pituitary deficiencies (van Tijn *et al*. 2008). A prolonged TRH test with a normal TSH increment may enable indirect assessment of TSH bioactivity, quantified by the rise in fT_3_ and fT_4_ levels at 120 min after TRH injection (Yamada & Mori 2008). However, although this information may help define the mechanism of CCH, further studies are needed to determine normal thyroid hormone responses at these timepoints. In isolated TSH deficiency, additional hormone measurements during TRH testing may enable differentiation between a likely *TSHB* mutation (preserved prolactin and α-subunit responses) and *TRHR* mutation (blunted responses) (Collu *et al*. 1997, Bonomi *et al*. 2001).

In the presence of central hypothyroidism, detailed investigation of the hypothalamo–pituitary axis is indicated as the majority of patients (up to 78% to 89%) will have additional pituitary hormone deficiencies (Mehta *et al*. 2003, Van Tijn *et al*. 2005). In a series of children with central hypothyroidism, GH deficiency was the most common associated hormone deficiency (89%), followed by ACTH (78%) and gonadotropin deficiency (46%), whilst posterior pituitary dysfunction was evident in a small percentage of patients (13%), all of who had SOD (Mehta *et al*. 2003). In this respect, MR imaging is important to detect structural pituitary abnormalities and midline or other CNS defects, and its results may guide genetic testing. Although patients with congenital central hypothyroidism may have a high prevalence of neonatal complications (hypoglycaemia, persisting jaundice, sepsis, seizures, feeding difficulties), the diagnosis of central hypothyroidism was only made in 28% during the neonatal period (Mehta *et al*. 2003).

**Figure 7:**
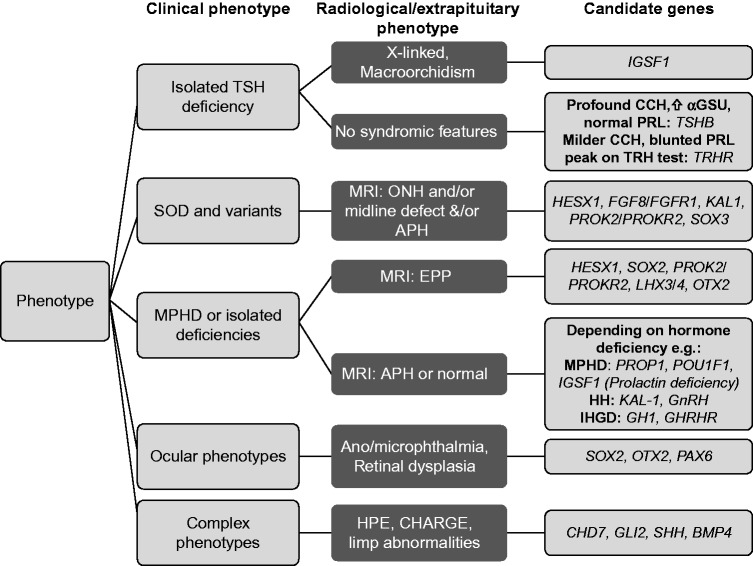
Proposed strategy for genetic testing in cases with CCH. MPHD, multiple pituitary hormone deficiencies; ONH, optic nerve hypoplasia; HPE, holoprosencephaly; HH, hypogonadotrophic hypogonadism; IHGD, isolated human growth hormone deficiency.

In specific cases, there is a role for targeted genetic screening, depending on the phenotype (Fig. 7). Patients with mutations in transcription factors (eg *PROP1, HESX1, SOX3) *require long-term surveillance for evolving ACTH and other pituitary hormone deficiencies. Conversely, identification of a genetic defect specific to the TSH biosynthetic pathway (*TSHB*, *TRHR*) enables reassurance that additional hormone deficits will not develop. Reports of families with *TRHR* or *IGSF1* mutations also highlight the fact that family screening following diagnosis in a young proband, may identify apparently healthy first, second or third-generation family members with hitherto undiagnosed central hypothyroidism. Untreated subclinical hypothyroidism is associated with adverse cardiometabolic risk, thus these individuals, who have overt central hypothyroidism may benefit from levothyroxine treatment both for cardiovascular health and for quality of life (Razvi *et al*. 2007, Singh *et al*. 2008, Bonomi *et al*. 2009).

## Treatment of central hypothyroidism

Data regarding treatment of central hypothyroidism primarily comes from adults with acquired hypothalamic–pituitary defects and definition of treatment targets remains challenging, since TSH cannot be used as a biomarker of euthyroidism. Furthermore, the negative feedback mechanism in central hypothyroidism may exhibit altered sensitivity to thyroid hormone, such that TSH concentrations above 1.0mU/l may reflect insufficient levothyroxine replacement (Carrozza *et al*. 1999, Ferretti *et al*. 1999, Shimon *et al*. 2002). Adequacy of T_4_ replacement is best assessed by fT_4_ measurement with the general consensus being that fT_4_ levels should be maintained in the mid-upper half of the normal range, although a lower target fT_4_ may be appropriate in the elderly (Ferretti *et al*. 1999, Slawik *et al*. 2007, Iverson & Mariash 2008, Koulouri *et al*. 2011). fT_3_ may be more sensitive in detecting overtreatment. Assessment of alternative biomarkers has identified that soluble interleukin 2 receptor (sIL2R) has some utility, but detects overtreatment more readily than undertreatment. SHBG, bone turnover markers, and cholesterol are all sensitive to changes in fT_4_, but the fact that they are also influenced by GH and gonadal status, renders them unreliable in combined pituitary hormone deficits (Ferretti *et al*. 1999).

It is imperative that cortisol deficiency is detected and treated before thyroid hormone replacement to avoid a hypoadrenal crisis. Once this is achieved, the drug of choice for treating central hypothyroidism is levothyroxine, with no current evidence to support T_3_/T_4_ combination therapy (Slawik *et al*. 2007). The dose needs to be tailored to the age and body weight of the patient, and must accommodate the influence of other hormone deficiencies or drugs. A reasonable target daily replacement dose of L-T_4_ would initially be 1.1 mcg/kg in patients over 60 years of age, 1.3 to 1.6 mcg/kg for younger adults and 50 to 100 μg/m^2^ per day in children (Ferretti *et al*. 1999, Slawik *et al*. 2007, Lania *et al*. 2008, Koulouri *et al*. 2011). In children, full replacement should be started immediately and monitored every 2 to 4 weeks initially whereas more cautious dosing may be necessary in the elderly or those with cardiac morbidities. Dose requirements may increase with concomitant oestrogen replacement which increases thyroid hormone binding proteins, and GH replacement, which can also unmask central hypothyroidism (Arafah 2001, Porretti *et al*. 2002).

## Future directions

The molecular basis for CCH remains to be ascertained in the majority of cases, raising aetiological questions in this subset. Candidate genes identified from murine models have been helpful in elucidating the basis of combined pituitary hormone deficiencies, and there are additional null mouse models of central hypothyroidism, which implicate other genes as potential candidates for human CCH. Such models include Trh −/− mice which exhibit central hypothyroidism with elevated TSH and hyperglycaemia and glycoprotein α subunit (Cga/Gsu) −/− null mice in whom TSH, T_4_, FSH and LH are undetectable (Yamada *et al*. 1997, Stahl *et al*. 1999). Additionally, despite the critical role of *GATA2 *and *PITX1* in the development of thyrotropes in murine models, no mutations have as yet been reported in patients with CCH. Perturbed thyroid hormone action and disruption of normal thyroid hormone-mediated negative feedback in the hypothalamus and pituitary may also cause CCH thus mice expressing a mutant NCOR1 corepressor exhibit central hypothyroidism due to a reset hypothalamic–pituitary thyroid axis (Astapova *et al*. 2011). In affected humans, studies using exome sequencing may also help elucidate less readily predictable causes.

Amongst the known genetic causes of CCH, the function of IGSF1 remains poorly understood and further *in vitro* and *in vivo* studies are required to delineate its mechanistic role in the anterior pituitary and its effects on testicular growth. Although current data suggest it is required for adequate TRHR mRNA expression, the expression of IGSF1 in all cells of the POU1F1 lineage and observed abnormalities of other hormone levels (GH, PRL, Testosterone) in deficient humans suggest that its role is more complex. Other cell surface immunoglobulin superfamily members bind specific antigens and interact with other receptors, such that a role for IGSF1 in paracrine signaling in the pituitary is plausible (Joustra *et al*. 2013*a*,*b*, Tajima *et al*. 2013). Prospective follow up of patients with *IGSF1* mutations will help define the natural history of the disease, addressing questions such as whether central hypothyroidism can evolve in carrier females. Additionally, genetic and environmental modifiers of the IGSF1-deficiency phenotype within individual kindreds have yet to be determined.

Long-term outcome studies in congenital central hypothyroidism are required, in particular to formally assess neuropsychological outcomes as a means of assessing adequacy of current diagnostic and treatment algorithms. Identification of robust biochemical or physiological biomarkers of thyroid hormone action will also help in diagnosis or treatment monitoring.

## Take home messages

- CCH has a higher incidence than previously thought (1:16 000), and delayed diagnosis may result in neurodevelopmental delay.
- CCH will evade diagnosis on TSH-based screening programmes, therefore a high index of suspicion is needed for prompt detection. The mainstay of biochemical diagnosis is the presence of subnormal T4 with inappropriately normal/low TSH, after exclusion of assay interference.
- CCH may be isolated or occur as a component of combined pituitary hormone deficiencies (majority of cases). Genetic ascertainment may predict evolution of other hormone deficits and enable prompt diagnosis and treatment of affected siblings.
- In combined pituitary deficits, phenotypes can be highly variable, and can evolve, therefore careful ongoing assessment is required in these patients.
- Family screening in cases with IGSF1 or TRHR mutations is important to identify apparently healthy relatives with hitherto undiagnosed central hypothyroidism who may benefit from levothyroxine treatment.
- Levothyroxine is an effective treatment for CCH; fT4 levels should be maintained in the mid-upper part of the normal range.
- Cortisol deficiency should be corrected before initiation of levothyroxine and the clinician should be alert to the fact that other hormone replacements (GH, oestrogen) may alter levothyroxine requirements.

## Figures and Tables

**Table 1:** Endocrine, Neuroradiological and extrapituitary manifestations of mutations in genes implicated in CCH in humans, and in the corresponding knockout mouse model

**Gene with mutation**	**Inheritance**	**Hormone deficits**	**Additional features**	**MRI**	**Mouse model**
TSHB	AR	TSH	−	E, N	−
TRHR	AR	TSH	−	N	*Trhr1* ^−/−^: N TSH, ⇓ T_4_, ⇓T_3_, ⇓PRL
Isolated TSH Deficiency or combined pituitary hormone deficiency					
IGSF1	XL^a^	TSH±PRL, GH (transient)	Macroorchidism (males) Ovarian cysts (females)	N	*Igsf1* ^Δ^ ^exon1 ^(male hemizygous):⇓TSH, ⇓T_3_, ⇓TRHR mRNA, normal pituitary PRL
Combined pituitary hormone deficiency					
POU1F1	AR, AD	GH, TSH, PRL	−	APH	*Pou1f1*dw/dw (Snell dwarf): ⇓TSH, ⇓PRL, ⇓GH, Dwarfism
PROP1	AR	GH, TSH, LH, FSH, PRL, evolving ACTH deficiencies	−	APH (may be transient), N, E	*Prop1* ^−/−^: ⇓TSH, ⇓GH, ⇓LH, ⇓PRL, ⇓FSH, Pituitary hypoplasia, dwarfism, hypogonadism
Specific Syndrome					
HESX1	AR, AD	Panhypopit GH and evolving TSH, ACTH, LH/FSH deficiency	Septo-optic dysplasia and its variants	APH, EPP, ACC, ONH	*Hesx1* ^−/−^: Anterior CNS defects, pituitary dysplasia, anopthalmia, defective olfactory development, bifurcations in Rathke's pouch.
LHX3	AR	GH, TSH, LH, FSH, PRL (ACTH)	Limited neck rotation, short cervical spine, sensorineural deafness	APH, N, E	*Lhx*3^−/−^: Absent TSH, GH, LH, PRL, pituitary hypoplasia, lethal
LHX4	AD	GH, TSH, ACTH, variable gonadotrophin deficiencies	Cerebellar abnormalities	APH, EPP	*Lhx4* ^−/−^:⇓TSH, ⇓GH, ⇓LH, ⇓PRL, ⇓POMC, pituitary hypoplasia, lethal
SOX3	XL	GH,TSH, ACTH, LH, FSH	Variable mental retardation	APH, EPP Persistent craniopharyn-geal canal	*Sox3* null (homozygous /hemizygous): Variable phenotype. More severe with craniofacial abnormalities, midline CNS defects ⇓TSH, ⇓GH, ⇓LH, ⇓FSH
OTX2	AD	GH, TSH, ACTH, LH, FSH	Uni/Bilat. Anophthalmia Retinal dystrophy	N, APH, EPP	*Otx2* ^−/−^:Absent forebrain and midbrain, lethal

E, Enlarged; N, Normal; APH, Anterior pituitary hypoplasia; EPP, Ectopic posterior pituitary; ACC, Agenesis of corpus callosum; ONH Optic nerve hypoplasia; TSH thyroid-stimulating hormone; LH, luteinizing hormone; FSH, follicle-stimulating hormone; ACTH, Adrenocorticotrophic hormone; Panhypopit.; Panhypopituitarism, AR, Autosomal recessive; AD, Autosomal Dominant; XL, X-linked, −/− homozygous null. References: Rabeler *et al*. (2004), Zeng *et al*. (2007). Sun *et al*. (2012), Camper *et al*. (1990). Nasonkin *et al*. (2004), Dattani *et al*. (1998), Sheng *et al*. (1996), Sheng *et al*. (1997), Rizzoti *et al*. (2004) and Acampora *et al*. (1995)

a But 1/3 females affected.
